# A Case Report and Literature Review of Gastric Stump Carcinoma: An Uncommon Entity Following the Billroth II Procedure

**DOI:** 10.7759/cureus.52354

**Published:** 2024-01-16

**Authors:** Nida Ansari, Mark Munairji, Gabriel Melki, Walid Baddoura

**Affiliations:** 1 Internal Medicine, St. Joseph's Regional Medical Center, Paterson, USA; 2 Gastroenterology, St. Joseph's Regional Medical Center, Paterson, USA

**Keywords:** anastomose, bile acid, gastro-jejunostomy, gastric tumor, billroth 2

## Abstract

Gastric stump carcinoma is a rare phenomenon and could occur in individuals after a distal gastric resection. Regardless of the surgical approach, it can lead to certain complications. However, the Billroth II gastrojejunostomy procedure has been noted to have some specifically interesting complications due to the anatomical changes it triggers. These changes, such as bacterial overgrowth and enterogastric reflux, can cause metaplasia. We discuss a case of an 81-year-old male with a history of peptic ulcer disease (PUD) status post-Billroth II gastrojejunostomy 30 years prior who presented with a four-day history of bright red blood per rectum. On esophagogastroduodenoscopy (EGD), he was found to have friable, ulcerated mucosa at the anastomosis site. Biopsy results revealed CDX2-positive cells, indicating gastric adenocarcinoma. Although it is well-known that the anatomical changes of gastrojejunostomy will undoubtedly change the microbiome of the stomach, physicians should also be mindful of the more feared complications such as gastric stump carcinoma.

## Introduction

Gastrojejunostomy is a surgical procedure that involves creating an anastomosis between the stomach and the jejunum [1]. There are various indications for this, such as bariatric weight loss surgery, bypass obstructions, and reconstruction after gastric resection [1]. As with most procedures, it has its potential complications, one of which involves intestinal contents being refluxed into the stomach and even the esophagus [1]. Billroth II procedures have been implicated more than Billroth I procedures in terms of causing complications [2]. Gastric stump carcinoma is believed to result from enterogastric reflux, among other causes [3]. Bile acids can induce changes in the epithelium [4]. It has been implicated as a carcinogen in colon cancer but has been seen to cause similar cell membrane changes throughout the gastrointestinal (GI) tract [4]. There has been a higher incidence of colon cancer in Western countries due to the increased consumption of high-fat diets [4]. While data in the literature on the implication of bile acids in the stomach is still expanding, it is believed that bile acids, combined with Heliobacter pylori infections, have a synergistic effect on metaplasia [4].

## Case presentation

An 81-year-old Hispanic male with a past medical history of hypertension and peptic ulcer disease (PUD) status post-Billroth II gastrojejunostomy 30 years ago presented to the emergency department (ED) with the chief complaint of bright red blood per rectum for four days. The patient denied nausea, vomiting, abdominal pain, diarrhea, or constipation. He also denied any prior episodes of hematochezia or melena or any non-steroidal anti-inflammatory drugs (NSAIDs) use. In the ED, vitals revealed blood pressure of 141/78 mm Hg, heart rate of 70 beats per minute, respiratory rate of 16 breaths/minute, and 100% saturation on room air. The physical exam was positive for hyperactive bowel sounds. The labs in the ED were as follows - blood urea nitrogen: 27 mg/dL, creatinine: 1.37 mg/dL, hemoglobin: 8.6 g/dL, mean corpuscular volume (MCV): 97 fL, and platelets: 114 K/mm^3^. 

The patient was admitted for further monitoring and treatment. He was started on IV fluids and IV pantoprazole infusion. The hemoglobin was monitored every six hours. he was placed on a clear liquid diet and given a polyethylene glycol-based bowel preparation. The following day, the patient was noted to have a hemoglobin of 6.6 g/dL and was transfused two units of packed red blood cells. Anemia workup revealed low vitamin b12 and low iron with a level of 38 mcg/dL, elevated ferritin at 431 mg/dL, total iron binding capacity of 294 mcg/dL, and iron saturation of 13%.

**Figure 1:**
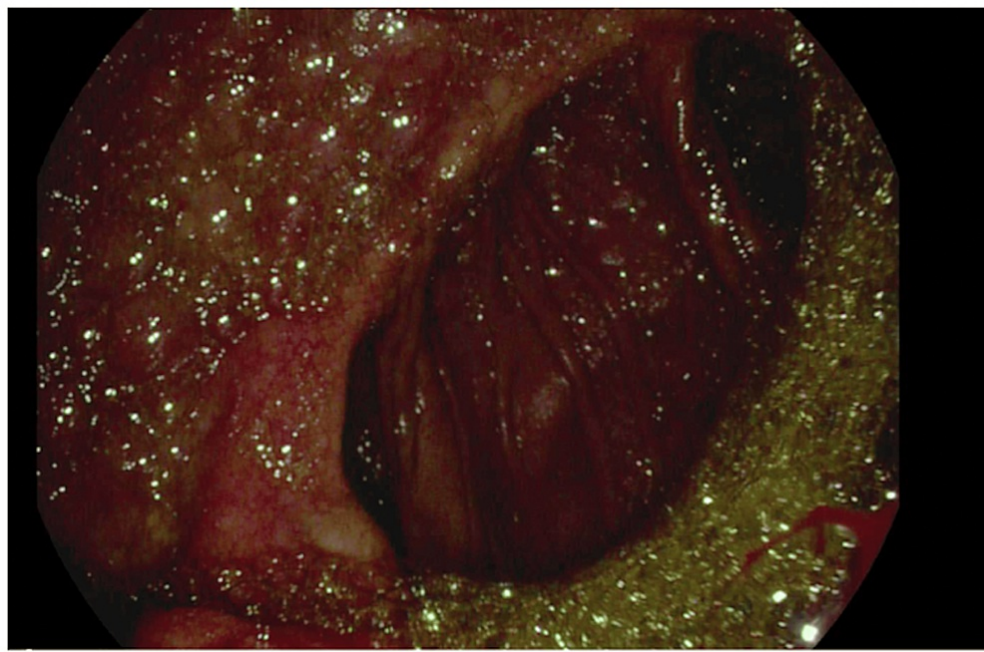
Gastrojejunostomy anastomosis site as seen on EGD The image shows edema, erosion, erythema, and ulceration EGD: esophagogastroduodenoscopy

**Figure 2:**
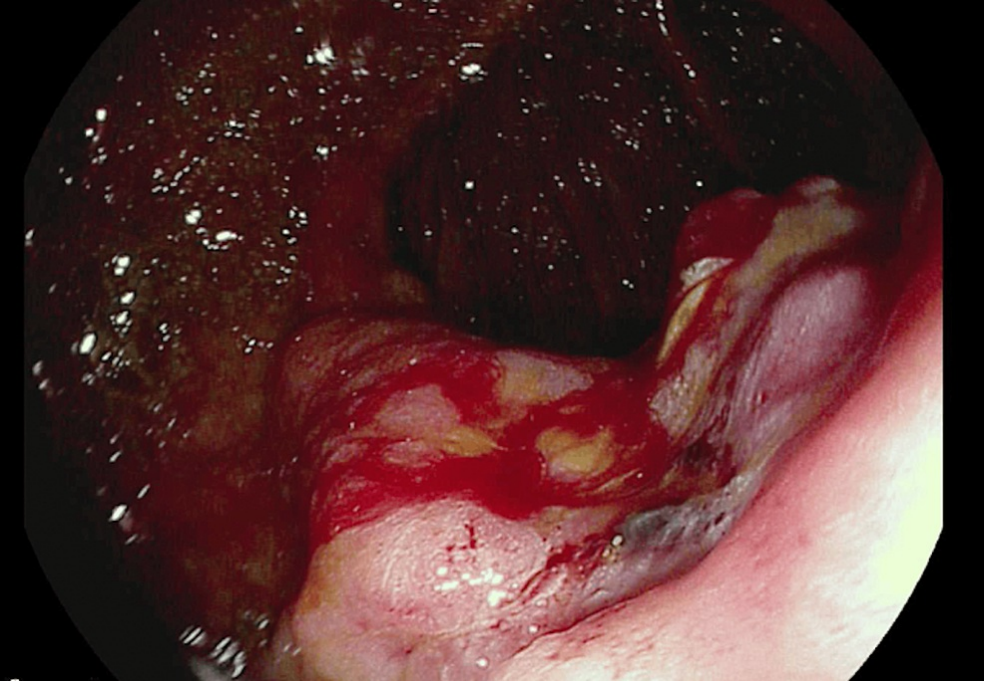
Gastrojejunostomy anastomosis site shows some minimal bleeding after biopsy with cold forceps

The patient underwent an esophagogastroduodenoscopy (EGD) and colonoscopy the following morning. The colonoscopy was significant for non-bleeding diverticulosis, grade I internal hemorrhoids, and one 3-mm polyp in the transverse colon. The EGD was significant for bleeding ulcerated mucosa friable at the Billroth II gastrojejunostomy anastomosis site, as seen in Figures 1-2. The patient’s renal function improved, and hemoglobin remained stable. He was optimized for discharge with outpatient follow-up.

The biopsy results from the EGD revealed poorly cohesive adenocarcinoma with many signet ring cells. Immunostains showed malignant cells positive for CDX2 and negative for GATA-3, Her2/neu, and Helicobacter pylori, which confirmed the diagnosis of poorly cohesive adenocarcinoma of the primary GI tract, likely intestinal-type gastric carcinoma.

## Discussion

The first recorded gastrojejunostomy was performed in 1881 by Anton Woelfer to bypass a cancer of the pylorus [1]. Roux-En-Y and Billroth II are the two most common techniques utilized for distal gastrectomy [2]. If the anastomosis between the stomach and jejunum does not include a separate pathway for pancreaticobiliary secretions, the procedure is referred to as a Billroth II [1]. The common complications are postoperative nausea and emesis; however, they are often short-lived [1]. Other complications include hemorrhage, deep vein thrombosis, embolus, anastomosed leak bowel obstruction, internal herniation, nutritional or micronutritional deficiency, dumping syndrome, bile reflux, and lastly, a marginal ulcer [1]. As He et al. stated, Billroth II anastomoses have a higher incidence of reflux esophagitis and remanent gastritis than Roux-en-Y anastomoses [2]. Due to the inability of reflux control, there is an increased disturbance of the gastric pH. Bile and pancreatic juice reflux harm the gastric remnant, as extensive gastritis has been seen in stomachs after the Billroth II procedure [2].

Post-Billroth II patients have been observed to have atrophic gastritis and a higher incidence of gastric carcinoma [2]. Due to the nature of the anastomoses, there is a higher potential for biliary and duodenal-pancreatic reflux into the remnant stomach and esophagus [2]. This reflux further increases the risk of malignancy [2]. Tersmette et al. reported an increased incidence of gastric remnant carcinoma 15-20 years after Billroth II surgery compared with Billroth I [5]. A mutant form of the p53 protein was detected in 10% of those patients [5].

Gastric stump carcinoma, also known as remnant gastric carcinoma, is a rare entity, accounting for only 2-6% of cases [3]. Defined as gastric carcinoma occurring post-resection, its time of onset has been debated in the literature, with some stating five years and others 10 years post-resection [3]. While the mechanism can be multifactorial, some prominent contributing factors include bacterial overgrowth, hypochlorhydria, and enterogastric reflux [3]. Bacterial overgrowth reduces dietary nitrates, allowing gastric epithelia's increased susceptibility to exposure to nitrosamines, a known carcinogen, to the gastric mucosa [3].

According to various studies, bile acids are carcinogenic [4]. Although colorectal cancer is the primary concern, it has also been linked to cancers in other parts of the gastrointestinal tract [4]. Populations with high incidences of colon cancer have been found to have higher fecal bile acid concentrations, and those with high dietary fat intake are at higher risk for gastrointestinal cancer [4]. With their high-fat diets, people in Western countries have higher incidences of colorectal cancer [4]. A retrospective study in 2022 found that serum total bile acids were elevated in patients undergoing operations for various gastrointestinal tumors compared to those without cancer, indicating a positive association between the two [4]. Additionally, individuals who have undergone cholecystectomies are at a higher risk of colon cancer due to the loss of the protective aspect of the gallbladder [4].

It has been proposed that bile acids disrupt cell membranes by promoting the generation of reactive oxygen species, which will further damage the DNA [4]. One study reported that mice fed diets higher in fat were found to have higher levels of 8-OHDG, which is used as a marker for oxidative damage, further implicating bile acids in carcinogenic actions [4]. Bile acids have also been implicated in apoptosis resistance [4]. Bernstein et al. stated that when colonic epithelial cells are repeatedly exposed to bile acid, the cells that were noted to evade cell death had gene expression changes, allowing them to develop apoptosis resistance [4]. Furthermore, this could be linked to the progression of colon cancer [4]. While most studies have shown a strong relationship between colorectal cancer and bile acids, they have been implicated in other malignancies as well [4]. Specifically, gastric cancer has been associated with bile acids by interacting with Helicobacter pylori infection [4]. Helicobacter pylori is a known carcinogen to the gastric biome; however, when coexisting with increased bile acids, it promotes epithelial dysfunction, leading to metaplasia. It is believed that the two have a synergistic effect on the development of malignancy [4]. The alteration of the pH environment secondary to enterogastric reflux allows for Epstein Barr Virus (EBV) infections [3]. EBV is an additional risk factor for gastric carcinoma and is implicated more commonly in Billroth II than in Billroth I [3].

## Conclusions

While the Billroth II procedure is not as common in Western countries compared to other techniques, clinicians need to be mindful of potential complications in patients who have undergone this type of procedure. The variety of changes that occur due to anatomical changes is plentiful, especially in those who have immigrated and adjusted to Western diets, which could induce metaplasia in the stomach. Bile acids are carcinogenic and, while more often implicated in colon cancer, clinicians must be aware of their carcinogenic effects throughout the GI tract.
